# Clinical Spectrum and Differential Diagnosis of Adult Thrombotic Microangiopathies: Real-World Experience from a Tertiary Referral Center

**DOI:** 10.3390/hematolrep18040049

**Published:** 2026-07-21

**Authors:** Nazlı Pelin Kırkayak, Gulsum Ozet, Simten Dagdas, Funda Ceran, Ihsan Ates

**Affiliations:** 1 Department of Internal Medicine, Ankara Bilkent City Hospital, Ankara 06800, Türkiye; dr.ihsanates@hotmail.com; 2 Department of Hematology, Ankara Bilkent City Hospital, Ankara 06800, Türkiye; gulsumozet@gmail.com (G.O.); simtendagdas@gmail.com (S.D.); ceranf@gmail.com (F.C.)

**Keywords:** thrombotic microangiopathies, thrombotic thrombocytopenic purpura, hemolytic-uremic syndrome, plasma exchange, adult

## Abstract

**Background/Objectives:** Thrombotic microangiopathies are rare, life-threatening hematological disorders characterized by microangiopathic hemolytic anemia, thrombocytopenia, and end-organ injury. This study was conducted in a setting where ADAMTS13 activity testing became available only from 2015 onward and complement-targeted therapy (eculizumab) had limited accessibility throughout most of the study period, conditions that shaped both diagnostic classification and treatment outcomes. Their clinical presentation, treatment response, and prognosis vary according to etiology, making early recognition and subtype classification clinically important. This study aimed to evaluate the etiological distribution, clinical features, treatment responses, and outcomes of adult patients with thrombotic microangiopathy at a tertiary-center real-world cohort. **Methods:** This retrospective cohort study included 47 adult patients (≥18 years) hospitalized with thrombocytopenia and microangiopathic hemolytic anemia (MAHA) in a nine-year period. Patients were classified as thrombotic thrombocytopenic purpura (TTP), hemolytic uremic syndrome (HUS), or secondary TMA based on clinical and laboratory evaluation. Demographic characteristics, clinical manifestations, laboratory parameters, treatments, and outcomes were analyzed. **Results:** The mean age was 45.3 ± 15.2 years, and 72.3% of patients were female. Primary thrombotic microangiopathy accounted for 74.5% of cases, including thrombotic thrombocytopenic purpura in 53.2% and hemolytic uremic syndrome in 21.2%; secondary thrombotic microangiopathy accounted for 25.5%. Hemodialysis was required in all patients with hemolytic uremic syndrome compared with 16% of those with thrombotic thrombocytopenic purpura. The complete response rate was 74.5%, and in-hospital mortality was 25.5%. In multivariable Cox regression analysis, treatment non-response and reduced post-treatment estimated glomerular filtration rate independently predicted mortality. **Conclusions:** Adult TMAs are characterized by considerable etiological and clinical heterogeneity, which makes differential diagnosis challenging, particularly in settings where access to contemporary diagnostic tests and targeted treatments is limited. In this cohort, in the absence of ADAMTS13 testing, TTP was the most frequent subtype, while treatment non-response and renal impairment emerged as the main factors associated with mortality. These findings emphasize the need for early clinical recognition and careful subtype-based differential diagnosis, which will reduce morbidity and mortality by permitting rapid initiation of pathophysiology-based appropriate interventions, i.e., PEx, immune suppression and caplacizumab for immune TTP and anti-complement therapy for aHUS, and limiting the inappropriate use of PEx with its complications, including sepsis.

## 1. Introduction

Thrombotic microangiopathies (TMAs) encompass a heterogeneous group of rare, life-threatening disorders characterized by microangiopathic hemolytic anemia (MAHA), thrombocytopenia, and ischemic end-organ injury due to microvascular thrombosis [1,2]. The annual incidence of TMAs is estimated at 10–15 cases per million population, though underestimation is common owing to diagnostic challenges, overlapping phenotypes, and underrecognition in clinical practice [1,3]. Historically viewed as distinct entities, TMAs are now classified as primary or secondary based on advances in molecular diagnostics and pathophysiology [2,4]. Primary TMAs include thrombotic thrombocytopenic purpura (TTP), Shiga toxin-associated hemolytic uremic syndrome (STEC-HUS), and complement-mediated hemolytic uremic syndrome (CM-HUS), which are mainly driven by ADAMTS13 deficiency, Shiga toxin-induced endothelial injury, and alternative complement pathway dysregulation, respectively [4,5,6]. Secondary TMAs are associated with triggers such as malignancy, pregnancy, autoimmune diseases, infections, transplantation, and drug exposure, often involving indirect endothelial damage and prothrombotic alterations [3,7]. Some patients classified as secondary TMA, particularly those with infection, pregnancy, or transplantation as the trigger, may actually have an underlying complement-mediated TMA that was simply brought on by that trigger. In these patients, treating the trigger alone may not be enough, and complement inhibition could offer additional benefit.

Clinical manifestations vary by etiology and organ involvement. Neurological symptoms predominate in TTP, while acute kidney injury (AKI) is prominent in HUS and transplant-associated TMAs [2,5]. The classic pentad (MAHA, thrombocytopenia, renal dysfunction, neurological impairment, fever) occurs in only a minority of cases, limiting its utility [8]. Early recognition and etiological classification are crucial, as treatments and outcomes differ across subtypes [4,9]. Therapeutic plasma exchange (TPE) has reduced TTP mortality from >90% to <20% with prompt initiation [4,9]. Supportive care is central for STEC-HUS, while eculizumab complement inhibition improves renal outcomes in CM-HUS [5,6].

Despite advances, secondary TMAs pose diagnostic and therapeutic challenges, with high mortality in cases involving malignancy, transplantation, or advanced renal involvement [3,7]. Recent studies identify infection, renal dysfunction, and cardiovascular complications as leading causes of death [3,10]. Long-term survivors often face persistent renal impairment, hypertension, neurocognitive deficits, and reduced quality of life [10,11].

However, real-world data on TMA etiology, treatment response, and mortality from tertiary-center cohorts remain limited [3,7,12,13]. Although advances in ADAMTS13 testing, complement diagnostics, and targeted therapies have transformed the contemporary management of TMAs, many real-world cohorts—particularly those spanning earlier periods—reflect a more pragmatic diagnostic approach based on clinical phenotype, laboratory patterns, organ involvement, and exclusion of alternative causes. Such cohorts remain informative for understanding how adult TMAs present in routine practice and which clinical features may assist early differential diagnosis when specialized testing is delayed or unavailable. ADAMTS13 activity testing was unavailable at our institution before 2015, and complement-targeted therapy was not readily accessible for most of the study period; both factors are essential context for interpreting the classification and outcomes reported here. Therefore, this study aimed to describe the clinical spectrum, etiological distribution, differential diagnostic features, treatment patterns, and outcomes of adult TMA patients managed over a nine-year period at a tertiary referral center.

## 2. Methods

### 2.1. Study Design and Setting

This retrospective single-centre cohort study was conducted at Ankara Numune Training and Research Hospital, a tertiary referral centre in Ankara, Turkey, in collaboration with Internal Medicine, Hematology, Nephrology, and Intensive Care departments. The study included patients admitted over a nine-year period (January 2010–December 2018).

### 2.2. Ethical Issues

The study was conducted in accordance with the Declaration of Helsinki and approved by the Clinical Research Ethics Committee of Ankara Numune Training and Research Hospital (approval date: 13 December 2018, decision no: 2381/2018). Because of the retrospective design and anonymized data, informed consent was waived by the ethics committee.

### 2.3. Study Population

The study population consisted of all adult patients (≥18 years) admitted to the hospital with thrombocytopenia (platelet count <150 × 10^3^/µL) and evidence of MAHA, defined as schistocytes on peripheral blood smear and elevated LDH [14], who were clinically suspected of having TMA. During the study period, 48 patients fulfilled these initial screening criteria. One patient who died within the first 24 h before diagnostic confirmation could be achieved was excluded. Thus, 47 patients were included in the final analysis. Patients were excluded if they had insufficient data for etiological classification, refused hospitalization or treatment, had a positive direct Coombs test or DIC at admission, or died before TMA diagnosis was confirmed. Follow-up data were available for 35 patients (74.5%) at 3 months, 33 patients (70.2%) at 6 months, and 33 patients (70.2%) at 12 months; the remaining patients were lost to follow-up or had incomplete records. Fibrinogen and D-dimer levels were recorded at admission for all patients to support exclusion of disseminated intravascular coagulation, which was applied as an exclusion criterion at study entry.

### 2.4. Data Gathering

Demographic characteristics, presenting symptoms, comorbidities, neurological findings, admission and discharge laboratory values, treatment modalities, and clinical outcomes were recorded using a standardized data extraction form. Laboratory variables included hemoglobin, platelet count, creatinine, eGFR, LDH, bilirubin, AST, and INR. Treatment data included TPE sessions, corticosteroid, rituximab, eculizumab, and hemodialysis use. Outcomes included platelet recovery; treatment response; hospital stay; discharge status; relapse; and 3-, 6-, and 12-month status.

### 2.5. Classification of Thrombotic Microangiopathy (Etiological Subgroups)

Patients were classified into etiological subgroups based on an integrated clinicopathological approach combining clinical presentation, laboratory findings, and available diagnostic tests. The diagnostic algorithm prioritized rapid clinical decision-making given the life-threatening nature of TMAs.

Thrombotic thrombocytopenic purpura (TTP): Patients with severely reduced ADAMTS13 activity (<10%) or those with high clinical probability of TTP (neurological predominance, minimal renal involvement, profound thrombocytopenia) were classified as TTP. Blood samples for ADAMTS13 activity were obtained prior to initiating therapeutic plasma exchange (TPE) in all cases. In patients with high clinical suspicion, TPE was initiated empirically while awaiting ADAMTS13 results.

STEC-associated HUS (STEC-HUS): Patients with ADAMTS13 activity >10% and presence of bloody diarrhea were classified as STEC-HUS. Shiga toxin testing, polymerase chain reaction, and/or stool cultures were performed when available to confirm the diagnosis.

Complement-mediated HUS (CM-HUS): Patients with ADAMTS13 activity >10%, absence of bloody diarrhea, no identifiable secondary trigger, and a renal-predominant clinical picture were classified as CM-HUS. Due to limited availability of complement genetic testing at our center during the study period, this classification was primarily clinical and based on exclusion of other etiologies.

Secondary TMA: Patients with ADAMTS13 activity >10% and an identifiable associated condition were classified as secondary TMA. Associated conditions included malignancy, hematopoietic stem cell transplantation, severe infection/sepsis, pregnancy-related disorders (preeclampsia, HELLP syndrome), and drug-induced TMA.

Because ADAMTS13 activity testing became available only in 2015 and complement genetic testing was limited, earlier cases were classified using clinical features and systematic exclusion of alternative diagnoses. Consequently, among the 47 patients, ADAMTS13 activity was measured in 13 (27.7%): 6 in the TTP group, 2 in the HUS group, 4 in the CM-HUS group, and 1 in the secondary TMA group. For patients treated before 2015 or in whom testing was not feasible, classification relied on clinical features and systematic exclusion of alternative diagnoses, following established diagnostic criteria. In all 13 tested patients, the ADAMTS13 result was concordant with the clinical classification (100%). Age (45.0 ± 10.6 vs. 45.4 ± 16.7 years; Welch *t*-test, *p* = 0.921) and sex distribution (Fisher’s exact test, *p* = 0.076) did not differ significantly between tested and untested patients.

### 2.6. Treatment and Response Assessment

Treatment decisions were individualized according to the suspected etiological diagnosis, clinical severity, organ involvement, and drug availability during the study period. Because the cohort covered 2010–2018, management reflected real-world institutional practice before widespread access to contemporary agents such as caplacizumab and before routine availability of comprehensive complement testing.

TTP: First-line therapy consisted of urgent TPE combined with corticosteroids, initiated as soon as TTP was clinically suspected. ADAMTS13 samples were obtained prior to the first TPE session. Patients with refractory or relapsing disease received pulse methylprednisolone and rituximab as second-line therapy. Rituximab was considered as second-line therapy in patients meeting criteria for refractory or relapsing TTP, specifically: platelet count <50 × 10^3^/µL after 5 days of TPE, failure to achieve clinical response within 7–20 days, platelet count not reaching >150 × 10^3^/µL, LDH not normalizing, or persistence of schistocytes on peripheral smear. Supportive care including transfusions and intensive care support was provided as clinically indicated. Caplacizumab was not available during the study period and was therefore not used. Rituximab was generally reserved for refractory or relapsing cases rather than routinely administered upfront, reflecting the treatment practice and access conditions of that period.

CM-HUS: First-line therapy was TPE, which was initiated promptly due to delays in eculizumab procurement in our setting. Patients with inadequate response to TPE received eculizumab when available. Eculizumab was considered when hemolysis persisted or renal function failed to improve after 3–5 days of TPE; in one additional patient with partial response, eculizumab was planned but could not be procured due to reimbursement constraints, and the patient was transferred to another center. Eculizumab and TPE were administered sequentially rather than concurrently in all cases in this cohort. In contemporary practice, eculizumab is increasingly favored earlier and, in some centers, as first-line therapy, in suspected CM-HUS; our sequential, TPE-first approach reflects the access constraints of the study period rather than current treatment preference.

Hemodialysis was initiated for renal replacement when clinically indicated. Eculizumab was not routinely available during most of the study period; therefore, TPE was used as an initial or bridging strategy in clinically suspected CM-HUS, although this approach does not reflect current preferred management where early complement inhibition is feasible.

STEC-HUS: Management was primarily supportive, avoiding antibiotics and antimotility agents. TPE was considered in severe adult cases with significant extrarenal manifestations or clinical deterioration. Hemodialysis was provided for renal replacement as needed. Corticosteroids were used in two STEC-HUS-labeled cases because of initial diagnostic uncertainty while ADAMTS13 results were pending.

Secondary TMA was managed by treating the underlying trigger and providing TPE or supportive care when clinically indicated. TPE and supportive care were provided in selected cases based on clinical judgment and disease severity [15].

Clinical response was defined as a platelet count >150 × 10^3^/µL and LDH < 1.5 times the upper limit of normal (ULN) after discontinuation of TPE. Clinical remission was defined as a clinical response persisting for more than 30 days after discontinuation of TPE. Exacerbation was defined as recurrence of thrombocytopenia or a rise in LDH within 30 days of the last TPE session, following an initial clinical response, requiring resumption of TPE. Relapse was defined as a platelet count <150 × 10^3^/µL occurring more than 30 days after discontinuation of TPE, requiring reinstitution of therapy. Refractory disease was defined as failure of the platelet count to rise or a platelet count persisting <50 × 10^3^/µL, with LDH remaining persistently above 1.5× ULN despite TPE and corticosteroid therapy [10].

### 2.7. Endpoints

The primary endpoint was the description of clinical symptoms and etiological distribution of TMA subtypes. Secondary endpoints included treatment response, in-hospital mortality, platelet recovery time, length of hospital stay, relapse rate, and long-term status (remission/relapse/death/lost to follow-up) at 3, 6, and 12 months after diagnosis. Relapse was defined as recurrence of thrombocytopenia and MAHA after achievement of complete remission. Follow-up data were obtained from outpatient clinic visits and hospital records.

### 2.8. Statistical Analysis

Statistical analyses were performed using SPSS version 20.0 (IBM Corp., Armonk, NY, USA). The normality of continuous variables was assessed using the Kolmogorov–Smirnov test. Normally distributed variables were expressed as mean ± standard deviation, whereas non-normally distributed variables were expressed as median with minimum and maximum values. Categorical variables were presented as frequencies and percentages.

Comparisons among TMA subgroups were conducted using one-way analysis of variance with Bonferroni post hoc testing for normally distributed variables and the Kruskal–Wallis H test with Dunn’s post hoc analysis for non-normally distributed variables. For two-group comparisons, the independent samples *t*-test or Mann–Whitney U test was applied as appropriate. Categorical variables were compared using the chi-square test or Fisher’s exact test.

Factors associated with in-hospital mortality were initially evaluated using univariable Cox proportional hazards regression analysis. Variables with *p* < 0.10 in univariable analysis were subsequently included in a multivariable Cox regression model using stepwise backward selection to identify independent predictors of mortality. Results are presented with hazard ratios (HR) and 95% confidence intervals (CI). Overall survival was analyzed using the Kaplan–Meier method, and survival curves were compared using the log-rank test. Within-group changes in laboratory parameters from admission to discharge were evaluated using paired *t*-test and Wilcoxon signed-rank test, as appropriate according to normality.

## 3. Results

A total of 47 patients were included in the final analysis. Primary TMA was predominant, with TTP being the most common subtype, followed by HUS and secondary TMA. The detailed etiological distribution is presented in Table 1.

The majority of patients were female, with a mean age in the mid-forties. Clinical presentations differed significantly among TMA subtypes (Table 2). The classic pentad of MAHA, thrombocytopenia, renal dysfunction, neurological impairment, and fever was present in only 10 of 47 patients (21.3%) overall, and in 6 of 25 TTP patients (24.0%), consistent with the well-established rarity of full pentad presentation in clinical practice (*p* = 0.999). Gastrointestinal symptoms were most frequent in STEC-HUS, while bloody diarrhea was characteristic of STEC-HUS (*p* = 0.001). Neurological symptoms were significantly more common in TTP and STEC-HUS compared with CM-HUS and secondary TMA (*p* = 0.001). Malignancy as a comorbidity was observed exclusively in secondary TMA (*p* = 0.037). Fibrinogen levels did not differ significantly across TMA subtypes and remained within or above the normal range in all groups (*p* = 0.093), arguing against occult disseminated intravascular coagulation in any subgroup. D-dimer and CRP levels differed significantly across subtypes (*p* = 0.027 and *p* = 0.016, respectively), with CM-HUS showing significantly higher values than TTP (D-dimer, *p* = 0.033; CRP, *p* = 0.024).

**Figure 1:**
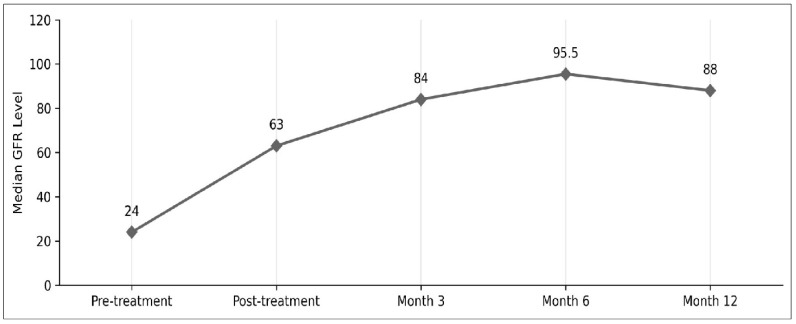
Longitudinal changes in estimated glomerular filtration rate during follow-up in patients with acute kidney injury.

Laboratory parameters demonstrated significant improvements during hospitalization, particularly in patients with TTP (Table 3). In TTP, hemoglobin and platelet counts increased significantly, accompanied by improvements in renal function and hemolysis markers. Platelet counts increased following treatment initiation and remained stable during long-term follow-up. In patients with acute kidney injury, eGFR improved significantly and remained elevated during follow-up (Figure 1). Secondary TMA showed significant improvements in platelet counts and hemolysis markers, while CM-HUS demonstrated significant decreases primarily in LDH and bilirubin levels.

Treatment characteristics and clinical responses varied among subtypes (Table 4). Patients with CM-HUS required significantly more plasmapheresis sessions compared with other subtypes (*p* = 0.005). Hemodialysis requirement differed markedly between groups; all patients with CM-HUS and STEC-HUS required renal replacement therapy, whereas the requirement was substantially lower in TTP and secondary TMA (*p* < 0.001). Rituximab was used in refractory TTP cases, and eculizumab was administered to one patient with CM-HUS.

Overall in-hospital mortality was 25.5%. Causes of in-hospital death according to TMA subtype are presented in Table 5. Clinical outcomes remained relatively stable across follow-up periods within each TMA subtype (Table 6). Loss to follow-up increased over time, particularly in HUS subtypes, which may have affected long-term outcome assessments.

**Figure 2:**
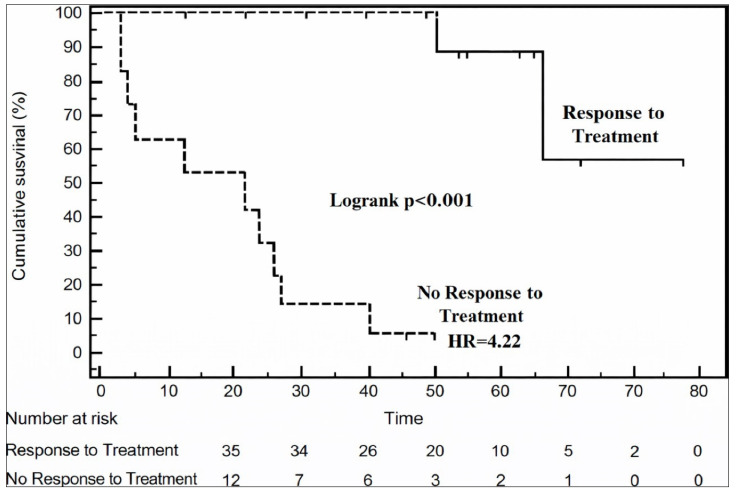
Kaplan–Meier survival curves according to treatment response in patients with thrombotic microangiopathy.

Kaplan–Meier survival analysis demonstrated significantly worse survival in non-responders compared with responders (log-rank *p* < 0.001) (Figure 2). Univariable and multivariable Cox proportional hazards regression analyses for predictors of mortality are presented in Table 7. In univariable analysis, significant predictors of mortality included chronic kidney disease, bleeding manifestations, myocardial infarction, absence of platelet recovery, treatment non-response, and several post-treatment laboratory parameters including reduced platelet count, elevated creatinine, reduced eGFR, elevated LDH, and elevated bilirubin levels. In multivariable analysis, treatment non-response (HR: 4.22, 95% CI: 1.85–9.61, *p* < 0.001) and reduced post-treatment eGFR (HR: 0.96 per 1 mL/min/1.73 m^2^ increase, 95% CI: 0.94–0.98, *p* = 0.002) emerged as independent predictors of mortality.

The association between reduced post-treatment eGFR and mortality was consistent across TMA subtypes: median (IQR) post-treatment eGFR was lower among non-survivors than survivors in TTP (30 [15–56] vs. 105 [92–118] mL/min/1.73 m^2^, *p* = 0.001), STEC-HUS/CM-HUS (17 [15–18] vs. 53 [40–71], *p* = 0.044), and secondary TMA (40 [30–48] vs. 65 [39–122], *p* = 0.283), although the secondary TMA comparison did not reach significance, likely reflecting limited statistical power (4 deaths among 12 patients). In a sensitivity logistic regression including post-treatment eGFR and TMA category, eGFR remained an independent predictor of mortality (*p* = 0.001) after accounting for subtype (*p* = 0.409), supporting an eGFR–mortality association that is not confined to the renal-predominant HUS phenotype.

## 4. Discussion

This study highlights the broad clinical spectrum and differential diagnostic complexity of adult TMAs in a tertiary-care real-world setting. TTP was the most frequently assigned subtype, followed by HUS phenotypes and secondary TMAs. However, considerable overlap was observed across clinical presentations, and the classic pentad was present in only a minority of patients. Neurological involvement was more suggestive of TTP, whereas renal-predominant disease and diarrheal prodromes supported HUS phenotypes. These findings should be interpreted in the context of limited access to ADAMTS13 testing before 2015, restricted complement diagnostics, and evolving therapeutic standards during the 2010–2018 study period. Therefore, the main value of this cohort lies not in validating a contemporary treatment algorithm, but in illustrating the diagnostic challenges and real-world classification patterns of adult TMAs. This diagnostic uncertainty mirrors findings from the French national TMA reference network registry, where the proportion of patients classified as TTP was only 4.5% among cases enrolled with systematic ADAMTS13 testing, compared with a substantially higher proportion when classification relied on clinical suspicion in earlier periods [16].

Treatment success varies by subtype. Plasma exchange is the mainstay for TTP, but timing and adjunctive therapy matter. Zheng et al.’s 2025 ISTH guidelines recommend urgent plasma exchange plus corticosteroids for TTP [17]; although these guidelines postdate our study period, the first-line approach we used was consistent with them, and we achieved an 84% complete response rate in TTP. De la Rubia et al. showed that rituximab is effective for relapsing TTP [18]. We used it in 12% of refractory cases. The low relapse rate observed (8%) should be interpreted cautiously: prolonged TPE courses before response was declared may have reduced early relapse, but incomplete long-term follow-up (60% at 12 months) limits detection of later relapses. Our TTP patients needed a median of 17 plasma exchange sessions. The relatively high number of TPE sessions likely reflects disease severity, delayed referral in a tertiary setting, and the absence of contemporary adjuncts such as caplacizumab during the study years. Of note, the definition of treatment refractoriness has evolved considerably; contemporary guidelines define inadequate response after approximately five plasma exchange sessions or 15 days of treatment, permitting earlier escalation to second-line agents such as rituximab. During the study period, patients typically received prolonged TPE before being classified as refractory, which may explain the high session counts observed in our cohort. In practice, non-response was typically declared only after 15–25 TPE sessions or 3–4 weeks of therapy, considerably later than the 5-session/15-day threshold now recommended, likely inflating both the apparent response rate and cumulative TPE-related complication burden. This differs from Bhagirath et al.’s HSCT-TMA review, where eculizumab achieved 80% hematologic response [19], reflecting limited eculizumab access rather than a difference in expected efficacy. One of our CM-HUS patients received eculizumab. For secondary TMA, we focused on treating the underlying cause, as Leisring et al. recommend [15]. All our HUS patients required dialysis, compared with 81.3% renal remission in Nguyen et al.’s more recent CM-HUS cohort, in which eculizumab was used as standard first-line therapy [10]—a treatment largely unavailable in our setting during the study period. This difference probably reflects disease severity and limited eculizumab access in our setting. Our results support Murphree et al.’s point that plasma exchange bridges the gap when eculizumab is not available [20]. The significantly higher number of plasmapheresis sessions observed in CM-HUS patients (*p* = 0.005) is likely attributable to the limited availability and procurement delays of eculizumab at our institution during the study period, which necessitated prolonged TPE as a bridging strategy rather than a definitive therapeutic choice. An important caveat applies to the interpretation of treatment response in CM-HUS. Plasma exchange and plasma infusion can transiently normalize platelet count, LDH, and haptoglobin—and, through replenishment of complement factor H and I in fresh frozen plasma, sometimes creatinine—without altering the underlying complement dysregulation. Patients may therefore meet laboratory response criteria while still progressing to end-stage renal disease or death. This is particularly relevant to our CM-HUS subgroup, where prolonged TPE bridged the absence of timely eculizumab access. In contemporary practice, early initiation of eculizumab in complement-mediated HUS is expected to substantially reduce TPE dependency and session burden. The absence of a significant fibrinogen difference across subtypes, with values remaining within or above the normal range throughout, supports the laboratory validity of our exclusion of disseminated intravascular coagulation and lends additional confidence to the classification of the secondary TMA group. The markedly higher D-dimer and CRP levels observed in CM-HUS compared with TTP further suggest a prominent inflammatory or prothrombotic burden in this subgroup, consistent with infection and inflammation as recognized triggers of complement dysregulation, and may partly explain the high sepsis-related in-hospital mortality observed in this group.

The management of TTP in this cohort should be interpreted in light of the historical period and resource constraints in which the patients were treated. Between 2010 and 2018, the accepted standard for acquired TTP was urgent therapeutic plasma exchange combined with corticosteroids, while rituximab was generally used for refractory or relapsing disease. This approach was consistent with the evidence and clinical practice available at that time. Current recommendations, including the 2025 ISTH update, support earlier use of rituximab and, when available, the addition of caplacizumab, an anti-von Willebrand factor nanobody shown to reduce TMA-related events, disease recurrence, and the need for repeated TPE sessions. However, caplacizumab was not approved or accessible at our institution during the study period. Therefore, its potential benefits, including shorter treatment courses and possibly fewer complications related to prolonged TPE, could not be reflected in our cohort. The relatively long TPE courses observed among our TTP patients, with a median of 17 sessions, should also be viewed in this context. They likely reflect both the severity of referred cases and the absence of early adjunctive therapies that are now expected to reduce dependence on prolonged plasma exchange. For this reason, direct comparison of our treatment protocol with current practice should be made cautiously. The outcomes reported in this study are better understood as real-world results shaped by the diagnostic and therapeutic limitations of the study period, rather than as deviations from current standards of care. Future prospective studies incorporating ADAMTS13 monitoring, caplacizumab, and complement genetic profiling are needed to define benchmarks that are more applicable to contemporary TMA management.

In TTP, plasma exchange replaces ADAMTS13 and removes autoantibodies, relieving neurological ischemia [20]. In CM-HUS, plasma exchange provides temporary complement control, but eculizumab blocks C5 more effectively [21]. For STEC-HUS, supportive care usually suffices because toxin clearance occurs naturally [22]. Although complete response was achieved in a substantial proportion of patients, this finding should be interpreted cautiously because treatment approaches were heterogeneous and not fully aligned with current targeted protocols. But, 23.4% of patients did not respond, showing that earlier escalation may be needed.

Treatment response and renal function predict survival. Non-responders face higher mortality risk. Peigne et al. found that renal failure and poor response drive mortality in ICU patients [23]. We found similar results. Non-response (HR: 4.22, 95% CI: 1.85–9.61) and reduced post-treatment eGFR (HR: 0.96, 95% CI: 0.94–0.98) independently predicted death. Yap et al. reported 31.6% mortality in Malaysia, mainly from sepsis [24]. Our 25.5% mortality rate was similar, and sepsis was also the main cause. Sepsis accounted for the majority of in-hospital deaths, underscoring infection-driven endothelial injury and microvascular thrombosis as key determinants of poor outcomes in this cohort. It is worth noting that the study period (2010–2018) predates contemporary advances in infection prevention and critical care management in TMA patients. More recent data suggest that earlier use of targeted therapies such as caplacizumab in TTP, combined with improved antimicrobial stewardship and intensive care protocols, may reduce sepsis-related mortality in current practice. The sepsis predominance observed in our cohort therefore likely overestimates the infection-related mortality burden expected in contemporary cohorts.

The vulnerability of TMA patients to sepsis likely reflects the immunosuppressive effects of prolonged corticosteroid use, central venous catheter placement for TPE, and underlying endothelial dysfunction. Non-responders had worse survival in our Kaplan–Meier analysis. Chen et al. found the same pattern in pregnancy-associated TMA [25]. Reassuringly, only one of our survivors developed chronic kidney disease. Bleeding and myocardial infarction also predicted mortality in our study, similar to Hwang et al.’s trauma-induced TMA analysis [26]. These systemic complications may be underreported outside ICU settings. Chart review identified catheter-related bloodstream infection in 4 of the 8 sepsis-related in-hospital deaths (50%), consistent with the immunosuppressive effects of prolonged corticosteroid use and the prolonged central venous catheter access required for repeated TPE sessions in this cohort. Because second-line therapies were less readily accessible during the study period, patients frequently underwent a higher number of TPE sessions before treatment was escalated or discontinued, which may have further increased catheter-related exposure and infection risk. Earlier escalation to second-line therapy, as increasingly practiced today, may reduce this risk in contemporary cohorts. Catheter dwell time and other infection surveillance metrics were not systematically coded as structured variables, however, precluding formal statistical analysis of this relationship.

Several limitations should be acknowledged. First, the retrospective single-centre design may have introduced selection bias, and the findings may not be generalizable to community hospitals or less severe TMA cases. Second, subgroup sizes were small, particularly for CM-HUS and secondary TMA, limiting the power to detect subtype-specific differences. Third, ADAMTS13 activity testing was unavailable before 2015 and was missing in most patients, although available results were consistent with clinical classification. Complement genetic testing was also not routinely available; therefore, some patients classified clinically may have had unrecognized complement-mediated or genetic predispositions. Fourth, treatment decisions, including the use of rituximab, eculizumab, and prolonged TPE, reflected real-world resource limitations and were not guided by a standardized protocol. Fifth, follow-up data were incomplete, which may have affected long-term remission, relapse, and survival estimates. Retrospective application of the PLASMIC score was not possible, as reticulocyte count, haptoglobin, and mean corpuscular volume were not systematically recorded for this cohort. Finally, the findings should be interpreted within the historical context of the 2010–2018 study period. ADAMTS13 testing was not available throughout the entire cohort, complement genetic testing and Shiga toxin testing were limited, and PLASMIC score-based decision-making was not systematically documented. These limitations may have caused some degree of etiological misclassification. In addition, caplacizumab was unavailable, rituximab was not routinely used as early adjunctive therapy in newly diagnosed immune TTP, and complement inhibition was delayed or unavailable in most suspected CM-HUS cases. Thus, treatment response and mortality data should be regarded as historical real-world outcomes under limited diagnostic and therapeutic access, rather than as results directly generalizable to contemporary TMA practice. Rather than proposing a new diagnostic or therapeutic paradigm, this cohort documents the diagnostic and therapeutic conditions that persisted in a resource-limited tertiary center up to a decade after ADAMTS13 testing and complement inhibition became standard of care in resource-rich settings.

In conclusion, adult TMAs are a clinically heterogeneous group of disorders, and accurate differential diagnosis remains difficult, especially in settings where ADAMTS13 testing, complement genetic analysis, and modern targeted therapies are not readily available. This real-world cohort shows that systematic clinical classification, based on combined hematological, renal, and neurological findings, can still provide meaningful etiological stratification under resource-limited conditions. The findings also indicate that treatment response and recovery of renal function are the main determinants of survival. Importantly, the treatment approaches used during the study period preceded several major advances in TMA care, including the routine use of caplacizumab in TTP and early complement inhibition in complement-mediated HUS. Therefore, the outcomes reported here should be viewed as historical real-world benchmarks rather than as results that would necessarily be expected with current management strategies. Overall, these findings emphasize the importance of early differential diagnosis for subtype-specific triage and support timely escalation to targeted therapies when they are available. Future prospective studies should include genetic profiling, serial ADAMTS13 monitoring, and contemporary treatment algorithms to establish outcome benchmarks that better reflect current clinical practice.

## Figures and Tables

**Table 1:** Etiological Distribution of Thrombotic Microangiopathy in the Study Population.

	Total Population n (%) (*n* = 47)
Primary TMA	35 (74.5)
TTP	25 (53.2)
Primary TTP (Idiopathic TTP)	23 (48.9)
Secondary TTP	2 (4.3)
STEC-associated hemolytic uremic syndrome	5 (10.6)
Complement-mediated hemolytic uremic syndrome	5 (10.6)
Secondary TMA (TMA-associated conditions)	12 (25.5)
Malignancy-associated TMA	4 (8.5)
Transplantation-associated TMA	1 (2.1)
Infection-associated TMA	5 (10.7)
Pregnancy-associated TMA (preeclampsia, HELLP syndrome, etc.)	2 (4.3)

Categorical variables are expressed as number (%). Abbreviations: HELLP: hemolysis, elevated liver enzymes, and low platelet count, STEC: Shiga toxin-producing Escherichia coli, TMA: thrombotic microangiopathy, TTP: thrombotic thrombocytopenic purpura. Secondary TTP (*n* = 2) is grouped under TTP within primary TMA because these cases were driven by severe ADAMTS13 deficiency (<10%) despite an identifiable secondary trigger.

**Table 2:** Demographic Characteristics, Clinical Presentation, and Comorbidities According to TMA Subtypes.

	TTP	STEC-HUS	CM-HUS	Secondary TMA	*p* Value
Sex					
Female	15 (60.0)	4 (80.0)	5 (100.0)	10 (83.3)	0.233
Male	10 (40.0)	1 (20.0)	-	2 (16.7)	
Age (years), mean ± SD	43.2 ± 13.0	54.2 ± 20.3	51.8 ± 10.0	43.3 ± 18.5	0.353
Presenting symptoms					
Nausea, vomiting, abdominal pain	13 (52.0)	1 (20.0)	4 (80.0)	4 (33.3)	0.002 *
Altered mental status	6 (24.0)	-	-	1 (8.3)	
Diarrhea	-	4 (80.0)	-	1 (8.3)	
Ecchymosis	1 (4.0)	-	-	1 (8.3)	
Purpuric rash	1 (4.0)	-	-	-	
Dyspnea	-	-	1 (20.0)	2 (16.7)	
Paresthesia of hands/feet	1 (4.0)	-	-	-	
Fatigue	-	-	-	3 (25.0)	
Gingival bleeding	3 (12.0)	-	-	-	
Diarrhea					
Absent	22 (88.0)	-	3 (60.0)	10 (83.4)	0.001 *
Present	3 (12.0)	5 (100.0)	2 (40.0)	2 (16.6)	
Bloody diarrhea	-	4 (80.0)	-	1 (8.3)	0.001 *
Watery diarrhea	3 (12.0)	1 (20.0)	2 (40.0)	1 (8.3)	
Comorbidities					
None	13 (52.0)	3 (60.0)	3 (60.0)	4 (33.3)	0.702
Present	12 (48.0)	2 (40.0)	2 (40.0)	8 (66.7)	
Diabetes mellitus	2 (8.0)	-	1 (20.0)	1 (8.3)	0.734
Hypertension	5 (20.0)	2 (40.0)	-	1 (8.3)	0.344
Malignancy	-	-	-	3 (25.0)	0.037 *
Systemic lupus erythematosus	1 (4.0)	-	-	1 (8.3)	0.991
Sjögren’s syndrome	1 (4.0)	-	-	-	0.999
Hypothyroidism	1 (4.0)	-	1 (20.0)	-	0.453
Familial Mediterranean fever	1 (4.0)	-	-	-	0.999
Chronic osteomyelitis	1 (4.0)	-	-	-	0.999
Chronic kidney disease	-	-	-	2 (16.7)	0.218
Neurological symptoms					
Absent	4 (16.0)	4 (80.0)	1 (20.0)	9 (75.0)	0.001 *
Present	21 (84.0)	1 (20.0)	4 (80.0)	3 (25.0)	
Altered mental status	11 (44.0)	1 (20.0)	2 (40.0)	3 (25.0)	0.633
Speech disturbance	-	-	1 (20.0)	-	0.206
Seizure	4 (16.0)	-	-	-	0.529
Paresthesia of hands/feet	5 (20.0)	-	-	-	0.271
Headache	1 (4.0)	-	1 (20.0)	-	0.447
ADAMTS13 activity					
Measured, n (%)	6 (24.0)	2 (40.0)	4 (80.0)	1 (8.3)	
<10%, n (%)	6 (24.0)	0	0	0	<0.001
>10%, n (%)	0	2 (40.0)	4 (80.0)	1 (8.3)	
Not measured, n (%)	19 (76.0)	3 (60.0)	1 (20.0)	11 (91.7)	
Fibrinogen (mg/dL), median (min–max)	306 (172–448)	213 (197–574)	521 (286–590)	285 (120–587)	0.093
D-dimer, median (min–max)	2580 (230–8000)	4075 (1970–20,000)	20,000 (2500–22,000)	2333 (1095–35,000)	0.027
CRP, median (min–max)	18 (1–243)	30 (25–71)	169 (39–262)	65 (3–265)	0.016

Categorical variables are presented as number (%). Continuous variables are expressed as mean ± standard deviation. * *p* < 0.05 was considered statistically significant. Abbreviations: ADAMTS13: a disintegrin and metalloproteinase with a thrombospondin type 1 motif, member 13, HUS: hemolytic uremic syndrome, SD: standard deviation, STEC: Shiga toxin-producing Escherichia coli, TMA: thrombotic microangiopathy, TTP: thrombotic thrombocytopenic purpura.

**Table 3:** Changes in Laboratory Parameters from Admission to Discharge According to TMA Subtypes.

Laboratory Parameters	TTP (Admission → Discharge)	*p* Value	HUS (Admission → Discharge)	*p* Value	Secondary TMA (Admission → Discharge)	*p* Value
Hemoglobin (g/dL)	7.8 ± 2.1 → 10.8 ± 1.9	<0.001 *	9.2 ± 1.3 → 9.3 ± 1.1	0.847	8.4 ± 2.0 → 9.1 ± 1.2	0.207
Platelet (×10^3^/µL)	15 (4–86) → 247 (15–424)	<0.001 *	72 (17–186) → 233 (71–325)	0.080	35 (10–94) → 156.5 (32–593)	0.021 *
Creatinine (mg/dL)	1.2 (0.6–8) → 0.8 (0.5–8.9)	0.032 *	3.2 (1.1–8.2) → 1.1 (0.4–5.5)	0.144	1.8 (0.5–4.5) → 1.4 (0.3–6.6)	0.158
GFR (mL/min/1.73 m^2^)	61 (8–130) → 96 (6–130)	0.002 *	18 (5–50) → 58 (13–119)	0.225	38 (12–170) → 49.5 (8–143)	0.136
LDH (U/L)	1307 (375–2516) → 229 (136–2225)	<0.001 *	824 (468–2070) → 330 (179–366)	0.043 *	1128 (334–3980) → 344 (147–1265)	0.002 *
Total bilirubin (mg/dL)	2.5 (0.6–10.8) → 0.5 (0.1–14.8)	0.012 *	1.3 (0.5–1.8) → 0.5 (0.4–0.9)	0.042 *	1.0 (0.4–26) → 0.6 (0.1–1.2)	0.015 *
Direct bilirubin (mg/dL)	0.5 (0.2–4) → 0.2 (0–7.5)	0.034 *	0.5 (0.3–0.6) → 0.2 (0–0.4)	0.041 *	0.4 (0–15) → 0.2 (0–0.6)	0.065
AST (U/L)	48 (13–201) → 21 (9–166)	0.001 *	49 (20–130) → 15 (10–35)	0.138	32.5 (17–739) → 22 (9–1523)	0.136
INR	1.2 (0.9–2.0) → 1.0 (0.9–2.7)	0.154	1.2 (1.0–1.3) → 1.0 (1.0–1.9)	0.715	1.1 (1.0–1.5) → 1.2 (0.9–1.9)	0.689

Continuous variables are presented as mean ± standard deviation or median (interquartile range), as appropriate. Within-group comparisons were performed separately for each TMA subtype. * *p* < 0.05 was considered statistically significant. Abbreviations: AST: aspartate aminotransferase, GFR: glomerular filtration rate, INR: international normalized ratio, LDH: lactate dehydrogenase, TMA: thrombotic microangiopathy, TTP: thrombotic thrombocytopenic purpura. *p* values reflect paired *t*-test (normally distributed variables) or Wilcoxon signed-rank test (non-normally distributed variables) for within-patient change from admission to discharge.

**Table 4:** Treatment Characteristics, Clinical Response, and Outcomes According to TMA Subtypes.

Clinical Characteristics	TTP	HUS	CM-HUS	Secondary TMA	*p* Value
Number of plasmapheresis sessions	15 (10–24)	7 (2–24)	25 (11–28)	6 (3–8)	0.005 *
Prednisolone use	21 (84.0)	2 (40.0)	4 (80.0)	7 (58.3)	0.109
Duration of prednisolone (months)	2.5 (1.5–3)	2.5 (2–3)	1.7 (1–2.5)	1.0 (0.8–1.5)	0.071
Pulse steroid therapy	3 (12.0)	-	1 (20.0)	2 (16.7)	0.922
Eculizumab use	-	-	1 (20.0)	-	0.207
Rituximab use	3 (12.0)	-	-	-	0.782
Hemodialysis	4 (16.0)	5 (100.0)	5 (100.0)	3 (25.0)	<0.001 *
Platelet recovery					
Absent	4 (16.0)	1 (20.0)	2 (40.0)	5 (41.7)	0.264
Present	21 (84.0)	4 (80.0)	3 (60.0)	7 (58.3)	
Time to platelet recovery (days)	9 (4–28)	6 (3–8)	5 (4–5)	6 (5–25)	0.056
Treatment response					
Response achieved	21 (84.0)	4 (80.0)	3 (60.0)	7 (58.3)	0.142
No response	4 (16.0)	1 (20.0)	1 (20.0)	5 (41.7)	
Partial response	-	-	1 (20.0)	-	
Length of hospital stay (days)	29 (3–63)	37 (4–48)	36 (31–55)	20 (5–75)	0.264
Clinical outcome at discharge					
Remission	19 (76.0)	4 (80.0)	3 (60.0)	7 (58.3)	0.447
Refractory disease	-	-	1 (20.0)	1 (8.3)	
Death	6 (24.0)	1 (20.0)	1 (20.0)	4 (33.3)	
Relapse					
Unknown	4 (16.0)	3 (60.0)	3 (60.0)	1 (8.3)	
No	19 (76.0)	2 (40.0)	2 (40.0)	10 (83.3)	0.112
Yes	2 (8.0)	-	-	1 (8.3)	

* *p* < 0.05 was considered statistically significant.

**Table 5:** Causes of In-Hospital Death by TMA Subtype.

Cause of Death	TTP n (%)	STEC-HUS n (%)	CM-HUS n (%)	Secondary TMA n (%)	Total n (%)
Sepsis/septic shock	4 (66.7)	—	1 (100.0)	3 (75.0)	8 (66.7)
Bleeding	—	1 (100.0)	—	—	1 (8.3)
Myocardial infarction	1 (16.7)	—	—	—	1 (8.3)
Hypotension during hemodialysis	1 (16.7)	—	—	—	1 (8.3)
Anaphylaxis	—	—	—	1 (25.0)	1 (8.3)
Total deaths	6 (24.0)	1 (20.0)	1 (20.0)	4 (33.3)	12 (25.5)

Percentages for cause-of-death rows reflect proportion within each subgroup’s deaths; percentages for total deaths row reflect proportion of patients within each subgroup. Fisher’s exact test: *p* = 0.579.

**Table 6:** Clinical Outcomes at 3 Months, 6 Months, and 1 Year According to TMA Subtypes.

	Outcome	3 Months n (%)	6 Months n (%)	1 Year n (%)	*p* Value
TTP	Remission	14 (56.0)	15 (60.0)	15 (60.0)	0.368
	Relapse	1 (4.0)	-	-	
	Death	6 (24.0)	6 (24.0)	6 (24.0)	
	Lost to follow-up	4 (16.0)	4 (16.0)	4 (16.0)	
STEC-HUS	Remission	2 (40.0)	1 (20.0)	1 (20.0)	0.355
	Relapse	-	-	-	
	Death	1 (20.0)	1 (20.0)	1 (20.0)	
	Lost to follow-up	2 (40.0)	3 (60.0)	3 (60.0)	
CM-HUS	Remission	2 (40.0)	1 (20.0)	1 (20.0)	0.348
	Relapse	-	-	-	
	Death	1 (20.0)	1 (20.0)	1 (20.0)	
	Lost to follow-up	2 (40.0)	3 (60.0)	3 (60.0)	
Secondary TMA	Remission	6 (50.0)	5 (41.7)	5 (41.7)	0.135
	Relapse	1 (8.3)	-	-	
	Death	4 (33.3)	5 (41.7)	5 (41.7)	
	Lost to follow-up	1 (8.3)	2 (16.7)	2 (16.7)	

Categorical variables are presented as number (%). Comparisons reflect differences in outcome distributions across follow-up periods within each subgroup. A *p* value < 0.05 was considered statistically significant. Abbreviations: STEC-HUS: Shiga toxin-producing Escherichia coli-associated hemolytic uremic syndrome; CM-HUS: complement-mediated hemolytic uremic syndrome; HUS: hemolytic uremic syndrome; TMA: thrombotic microangiopathy; TTP: thrombotic thrombocytopenic purpura.

**Table 7:** Cox Proportional Hazards Regression Analysis for Predictors of Mortality.

Variable	Univariable			Multivariable		
	HR	95% CI	*p*	HR	95% CI	*p*
**Demographics**						
Male sex	1.60	0.50–4.90	0.459	—	—	—
Chronic kidney disease	10.20	1.73–60.34	0.010	—	—	—
**Clinical features**						
Bleeding manifestations	3.78	1.19–12.07	0.025	—	—	—
Myocardial infarction	52.40	3.10–885.70	0.006	—	—	—
**Treatment factors**						
Platelet recovery	0.05	0.01–0.21	<0.001	—	—	—
Treatment non-response	4.54	2.14–9.64	<0.001	4.22	1.85–9.61	<0.001 *
Post-treatment labs						
Hemoglobin (per g/dL)	0.70	0.50–1.00	0.030	—	—	—
Platelet (per 10^3^/µL)	0.98	0.97–0.99	<0.001	—	—	—
Creatinine (per mg/dL)	1.31	1.09–1.57	0.004	—	—	—
eGFR (per mL/min/1.73 m^2^)	0.97	0.95–0.99	0.001	0.96	0.94–0.98	0.002 *
LDH (per 100 U/L)	1.10	1.05–1.15	<0.001	—	—	—
Total bilirubin (per mg/dL)	1.20	1.08–1.33	0.001	—	—	—

Model fit: −2 Log likelihood = 65.96; *p* < 0.001. * *p* < 0.05 was considered statistically significant. Abbreviations: CI, confidence interval; eGFR, estimated glomerular filtration rate; HR, hazard ratio; LDH, lactate dehydrogenase.

## Data Availability

Data supporting the findings of this study are available from the corresponding author upon reasonable request.

## Abbreviations

AKI	acute kidney injury
CI	confidence intervals
CM-HUS	complement-mediated hemolytic uremic syndrome
eGFR	estimated glomerular filtration rate
HR	hazard ratios
HUS	hemolytic uremic syndrome
MAHA	microangiopathic hemolytic anemia
STEC-HUS	Shiga toxin-associated hemolytic uremic syndrome
TMA	thrombotic microangiopathies
TPE	therapeutic plasma exchange
TTP	thrombotic thrombocytopenic purpura
